# Late vs. Early Preeclampsia

**DOI:** 10.3390/ijms262211091

**Published:** 2025-11-16

**Authors:** Maria Kariori, Vasiliki Katsi, Costas Tsioufis

**Affiliations:** 1st Department of Cardiology, Hippokration Hospital, Medical School, National & Kapodistrian University of Athens, 114 Vasilisis Sofias Avenue, 11527 Athens, Greece; vkkatsi@yahoo.gr (V.K.);

**Keywords:** preeclampsia, early onset, late onset, genetics, management strategies

## Abstract

Preeclampsia (PE) is a complex hypertensive disorder of pregnancy characterized by new-onset hypertension and proteinuria after 20 weeks of gestation. It is classified into early-onset (EOPE, <34 weeks) and late-onset (LOPE, ≥34 weeks) subtypes, which differ in their pathophysiology, clinical course, and maternal and neonatal outcomes. EOPE arises from abnormal placentation with inadequate spiral artery remodeling and impaired uteroplacental perfusion, whereas LOPE is mainly related to maternal cardiovascular and metabolic predisposition. This review integrates current molecular, immunological, and hemodynamic evidence distinguishing EOPE from LOPE, emphasizing recent insights into angiogenic imbalance (VEGF, PlGF, sFlt-1), oxidative stress, and immune modulation. It also summarizes evolving diagnostic and prognostic biomarkers and evaluates emerging therapeutic approaches, including gene therapy targeting placental dysfunction. By comparing mechanistic pathways and clinical implications, this review highlights how gestational age–specific pathogenesis may inform risk stratification, early detection, and precision-based management of PE.

## 1. Introduction

Pre-eclampsia (PE) constitutes a disorder of arterial hypertension during pregnancy, presenting the main characteristics of new-onset hypertension and proteinuria after the 20th gestational week, with several multisystem implications for both the mother and the fetus. Early-onset preeclampsia (EOPE) and late-onset pre-eclampsia (LOPE) are distinct clinical entities. They present different pathophysiological pathways and outcomes, and therefore require different management strategies. EOPE is a mixed disorder; thus, it is characterized by placental dysfunction and intrauterine growth restriction, resulting in adverse maternal and neonatal outcomes. It occurs before the 34th gestational week. LOPE is mainly considered a maternal disorder. Its main characteristics include a normal placenta, favorable maternal and neonatal outcomes at or after the 34th gestational week. This classification is representative not just of timing but also of various biochemical markers, genetic and environmental risk factors, prognosis, and clinical features [1,2,3]. Additionally, to classification by gestational age, preeclampsia is also clinically categorized according to disease severity based on the degree of hypertension, proteinuria, and involvement of target organs such as the liver, kidneys, and brain. Severe preeclampsia is characterized by markedly elevated blood pressure, end-organ dysfunction, and a higher risk of maternal and fetal complications. Importantly, severe cases may occur in both early- and late-onset forms, reflecting overlapping mechanisms of placental dysfunction and maternal endothelial injury. This review recognizes the mechanistic and clinical distinctions, providing the guidance for improved diagnostic accuracy, individualized management strategies, and prevention of pregnancy-related complications. 

### Definitions of Preeclampsia

There are five definitions of PE based on the finding of an additional feature (i.e., a maternal end-organ dysfunction, with or without uteroplacental dysfunction, depending on the definition) among women with chronic hypertension or in association with new-onset hypertension among other women. The diagnostic criteria for PE vary slightly among major international societies, emphasizing combinations of new-onset hypertension and maternal or uteroplacental dysfunction. These definitions are summarized in Table 1, together with specific blood pressure thresholds and associated clinical findings [4].

## 2. Pathophysiologic Mechanisms

**Figure 1:**
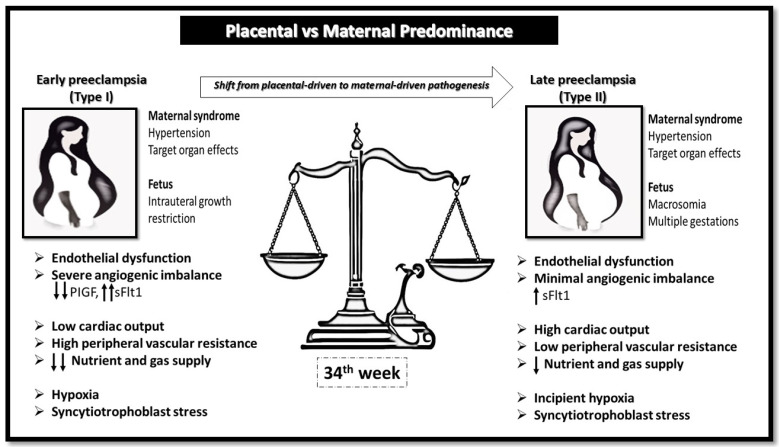
Conceptual representation of early-onset (Type I) and late-onset (Type II) preeclampsia as distinct but overlapping entities along a gestational continuum. The balance scale illustrates the shift in pathophysiological predominance between placental dysfunction (left) and maternal cardiovascular maladaptation (right), with the 34th week marking the clinical threshold separating early and late forms. Early-onset preeclampsia is primarily characterized by defective placentation, severe angiogenic imbalance (↓PlGF, ↑sFlt1), and fetal growth restriction, while late-onset preeclampsia is mainly associated with maternal cardiovascular factors, endothelial dysfunction, and increased long-term cardiovascular risk.

The differences between EOPE and LOPE refer not only to the onset of the disease but also to its pathogenesis. EOPE is mainly a disorder of abnormal placentation, which leads to hypoxia, oxidative stress, syncytial damage, and systemic endothelial dysfunction. In contrast, LOPE is more often a disorder of maternal cardiovascular adaptation, where women with pre-existing pathologies develop clinical disease later in gestation despite a largely normal placenta. As illustrated in Figure 1, these processes highlight the mechanisms underlying abnormal placentation and angiogenic imbalance that distinguish EOPE from LOPE. However, in cases first diagnosed ≥ 34 weeks without earlier assessments, the condition is classified as late-onset by definition; nevertheless, features such as severe fetal growth restriction, abnormal uterine artery Doppler indices, and a markedly elevated sFlt-1/PlGF ratio may indicate a predominant placental-insufficiency phenotype, guiding closer surveillance and expedited delivery planning [5,6,7,8,9].

There are three hypotheses for the pathophysiologic pathway of early- and late-onset preeclampsia: abnormal placentation, the contribution of angiogenic/antiangiogenic factors, and oxidative stress.

### 2.1. Abnormal Placentation

The PE onset is closely tied to inadequate trophoblast invasion, which triggers endothelial dysfunction and an exaggerated inflammatory response. After blastocyst implantation, trophoblast cells are among the first to differentiate, giving rise to both cytotrophoblast and syncytiotrophoblast lineages. Cytotrophoblasts then follow two main developmental pathways: the extravillous (invasive) and the villous (syncytial). In the extravillous pathway, cytotrophoblasts gradually invade the decidua basalis and the uterine wall, disrupting the endothelium and the tunica media of the spiral arteries [10,11,12], thereby converting them from high-resistance to low-resistance, high-flow vessels [10]. This procedure actually increases uteroplacental perfusion [11] and creates the proper conditions for a normal pregnancy to proceed.

In PE, this physiological remodeling of the myometrial spiral arteries does not occur properly, leaving them abnormally narrow when compared with those in uncomplicated pregnancies [13,14]. Therefore, blood supply to the fetus becomes restricted [10], due to impaired uteroplacental circulation [11], producing a placenta that is poorly perfused, ischemic, and hypoxic. These conditions promote ischemia–reperfusion injury and oxidative stress [10], resulting in fetal growth restriction [11].

Histopathological findings in early-onset PE typically demonstrate poor trophoblastic invasion, incomplete spiral artery remodeling, and increased fibrinoid necrosis, which is associated with impaired placental perfusion and hypoxia [12,15,16]. However, in placentas from late-onset PE pregnancies, minimal structural abnormalities or focal lesions of maternal vascular malperfusion are present. This characteristic reflects predominant maternal endothelial dysfunction rather than placental insufficiency [5,6,17]. From an imaging perspective, gross placental morphologic features on ultrasound (e.g., thickening or lobulation) are nonspecific; clinical assessment relies primarily on Doppler interrogation of the uterine–placental–fetal circulation [7,18,19,20]. Clinically, EOPE is more often associated with fetal growth restriction and abnormal uterine artery Doppler flow. In contrast, LOPE presents with normal placental perfusion and features of maternal metabolic or cardiovascular stress [7,19,20].

In healthy pregnancies, villous cytotrophoblasts remain at the basal membrane beneath the syncytiotrophoblast, differentiate later, and fuse with it to form the syncytium. However, levels of both apoptotic debris [13] and anti-angiogenic factors [21] are elevated in PE. Therefore, hypoxia of the placenta releases large amounts of vasoactive factors, disrupts syncytial structure, and finally leads to endothelial dysfunction [22]. Persistent hypoxia further exacerbates oxidative stress, increases apoptosis and necrosis, and promotes the shedding of placental debris [23]. Such placental abnormalities are more strongly associated with EOPE.

However, LOPE is believed to result mainly from maternal microvascular disorders, such as chronic hypertension, or genetic predisposition [24]. Therefore, EOPE is usually linked with fetal growth restriction, poor spiral artery invasion, abnormal placental blood flow, and elevated vascular resistance, while in LOPE, fetal growth restriction and spiral and umbilical artery blood flow abnormalities are absent [25]. Placental structural abnormalities also characterize EOPE. In LOPE, the placenta is almost identical to that of healthy pregnancies [7]. Various molecular markers of placental dysfunction have been investigated in women who later develop PE, and identifying these may allow earlier detection and better management. The following section reviews key angiogenic and placental biomarkers.

### 2.2. Maternal Cardiovascular Adaptations During Pregnancy

Pregnancy requires a multisystem maternal adaptation to meet the burgeoning fetal demand. The maternal cardiovascular response in a healthy subject is similar to the changes that are exercise-induced. These changes include physiological hypertrophy, improved function, and enhanced metabolism. Unlike chronic conditions, pregnancy does not typically lead to deleterious alterations despite its continuously growing demands [26]. Gestational cardiac hypertrophy is activated by pregnancy hormones, including progesterone, which reduces PVR and contributes to functional changes [27,28]. Syncytiotrophoblast estrogens and progesterone promote vasodilation of the uterine arteries and other placental bed vascular adaptations [29,30]. These changes, along with alterations in the renal and pulmonary systems, increase CO and blood volume while reducing PVR. Several echocardiographic parameters have been used to assess maternal cardiac adaptations in normal and preeclamptic pregnancies. In pregnancy, there is volume overload of the venous compartment, leading to cardiac overload, even without PE or an abnormal placenta [31]. The increased risk of PE associated with obesity or metabolic syndrome, which increases CO and intravascular volume, may be partly driven by this further volume overload [8,19,32]. Intravascular overload triggers an endothelial response, leading to the release of systemic inflammatory mediators, reactive oxygen species, serum microRNAs, cell-free DNA, and extracellular vesicles [20,33]. PlGF has been found to play an essential role in cardiovascular remodeling in both mice and humans [34,35]. Studies using a PlGF knock-out murine model demonstrated that PlGF deficiency is associated with a preeclampsia-like syndrome, characterized by higher systolic blood pressure, lower CO, and hypertrophic kidneys with glomerular pathology compared with controls [35]. Thus, hypertension presents along a spectrum of unbalanced states of CO and vascular resistance, which may vary from resistance dominance to volume dominance, or appear as a combination form [31]. In humans, PlGF levels were studied at mid-pregnancy, and the cohort was followed up at 6 and 9 years after delivery. Patients with PlGF in the lowest quartile at mid-pregnancy displayed long-term markers of abnormal cardiac remodeling, including enlarged aortic root and left atrial diameters and left ventricular mass, as well as higher blood pressure, even among those with uncomplicated pregnancies [32]. More recently, Giorgione et al. demonstrated a correlation between sFlt-1, PlGF, and their ratio and maternal echocardiographic indices in patients with hypertensive disorders of pregnancy. Their results lend support to the notion that angiogenic imbalance during pregnancy is associated with adverse maternal cardiovascular outcomes in pregnancy and beyond [36].

Pregnancy without placental PlGF-modulated remodeling produces measurable long-term injury to the maternal cardiovascular system. These studies in humans and murine models highlight that low PlGF, even in the absence of clinical PE, has long-term deleterious effects, further supporting the imperative role of placental PlGF in gravid remodeling of the cardiovascular system [5,34,35]. Indeed, maternal cardiac maladaptation has been observed across various parameters. In a study comparing uterine artery Doppler and echocardiographic measures among preeclamptic women and normotensive controls, the EO preeclamptic group showed reduced ventricular wall thickness and diameters, lower CO and apparent underfilling, and signs of pressure overload and concentric hypertrophy. The echocardiographic findings of women with late-onset disease included dilated ventricles, high CO, ventricular hypertrophy, and signs of overfilling without pressure overload. Early-onset and late-onset PE also differed in PVR, with higher PVR in early-onset disease and lower PVR in late-onset disease [6]. It has been shown that increased Doppler indices of the ophthalmic arteries in the late first trimester are predictive of the development of PE [9,37,38], and that those measured in the third trimester are predictive of the development of PE within the next 3 weeks [9]. Considering the pervasive maternal cardiovascular adaptations that occur during pregnancy and the disorders in these adaptations observed in PE, it is unsurprising that they extend to divergent adaptations in the peripheral vasculature, such as the ophthalmic arteries.

### 2.3. Angiogenic and Antiangiogenic Factors

Although many pro- and antiangiogenic molecules have been implicated in placental vascular development, vascular endothelial growth factor (VEGF) and placental growth factor (PlGF) are among the most important factors in the pathogenesis of PE. Both belong to related families and are involved in trophoblast proliferation and implantation. stabilizes endothelial cells within the mature vessels [39]. PlGF promotes angiogenesis triggered by conditions such as inflammation and hypoxia [39]. Therefore, the VEGF/PlGF signaling pathway is central to the pathophysiology of PE, as supported by numerous studies. Several investigations have demonstrated decreased levels of these factors in PE [3,39,40,41]. Notably, PlGF concentrations during the second and third trimesters tend to be higher in women with LOPE compared to those with EOPE [2,18,22,42,43]. However, a recent study found that median first-trimester PlGF levels cannot serve as a reliable predictive marker of PE.

Urinary PlGF levels also appear not to differ between women who later develop EOPE versus LOPE [44]. VEGF-A levels between pre-delivery and postpartum samples in either early- or late-onset groups, and postpartum PlGF levels, did not differ significantly [43]. In addition to the angiogenic role of VEGF and PlGF, antiangiogenic factors are crucial contributors to PE pathogenesis. VEGF and PlGF normally bind to fms-like tyrosine kinase (Flt-1) receptors, which regulate the production of soluble Fms-like tyrosine kinase (sFlt1). Acting as an antagonist, sFlt1 prevents VEGF and PlGF from binding to their receptors. Placental-derived sFlt1 in maternal serum is linked to hypertension and, alongside endoglin, has been shown to induce PE-like symptoms in pregnant rats [22,24]. Elevated sFlt1 may underlie the maternal endothelial dysfunction characteristic of PE [45]. Placental expression of sFlt1 is upregulated in PE [46], and in some studies, its levels correlate with disease severity [43,47]. Increased maternal plasma concentrations of sFlt1 are observed 2–3 months before PE onset [48]. Both EOPE and LOPE are associated with altered sFlt1 levels, though the changes are typically more pronounced in EOPE [2,7,18,49,50]. Women with EOPE may exhibit up to 43-fold higher median sFlt1 levels compared with controls, while those with LOPE show about a 3-fold increase [2]. Nevertheless, some studies have not found significant differences between the two subtypes [22,50] due to variation in sample sizes, classification criteria, or gestational age at testing. Postpartum, sFlt1 levels remain elevated in EOPE but not in LOPE when compared with controls [43]. Recent work suggests that the sFlt1/PlGF ratio may be more predictive of PE than either marker alone [3,50,51], though no clear differences in this ratio have been reported between EOPE and LOPE [50].

Another receptor, tyrosine kinase with immunoglobulin-like and EGF homology domains 2 (Tie-2), expressed on endothelial cells, is vital for angiogenic remodeling and vessel stabilization [52]. In EOPE, lower levels of soluble Tie-2 were observed when compared to LOPE [53].

VEGFR-1, a high-affinity receptor for VEGF, contributes to its biological effects. A soluble splice variant, sVEGFR-1, sequesters VEGF and inhibits its activity. Elevated maternal plasma concentrations of sVEGFR-1 have been documented in PE [54], correlating with disease features such as proteinuria, platelet count, and clinical severity [55]. Both early- and late-onset PE show increases in sVEGFR-1 [56], but patients with EOPE typically present with earlier and more pronounced elevations [57]. sVEGFR-1 has been proposed as a better predictor for EOPE, with higher sensitivity and specificity compared to LOPE [53].

Endostatin, another antiangiogenic factor, inhibits endothelial proliferation and migration, inducing apoptosis [58]. Elevated maternal serum levels of endostatin have been reported in PE [59,60]. In comparative studies, it has been found that increased endostatin levels are more common in EOPE [61] further supporting the link between EOPE and abnormal placental angiogenesis.

Epidermal growth factor (EGF) and transforming growth factor-β (TGF-β) play opposing roles in trophoblast syncytialization; EGF promotes it [62], while TGF-β disrupts it [11]. Therefore, the balance between these two factors is rather important. In patients with PE, reduced EGF [63] and elevated TGF-β levels [64] have been recorded. EGF, particularly its heparin-binding isoform, also reduces hypoxia-induced trophoblast apoptosis [65], but its levels are diminished in PE [66]. While earlier studies found no differences in TGF-β1 between PE and healthy pregnancies [56,67], more recent work indicates higher concentrations in PE [68], and genetic predisposition for elevated TGF-β1 production has been noted [69]. However, no studies have yet compared TGF-β1 and EGF specifically between EOPE and LOPE.

Endoglin is a co-receptor for TGF-β1 and TGF-β2, which is expressed on endothelial cells and syncytiotrophoblasts [10,70,71]. Its soluble form (sEng) is markedly increased in women with PE [50], often preceding symptom onset [69]. In pregnant rats, sEng induces severe PE-like disease, including HELLP syndrome and fetal growth restriction [51]. However, findings on sEng levels in EOPE versus LOPE remain inconsistent: some studies report no difference [50,72], while others report higher levels in EOPE [17,43,53]. Another study found increases in both sEng and sFlt1 in the first trimester of women who later developed LOPE [49]. The predictive value of sEng thus remains uncertain, though combined analysis with sFlt1 appears more robust, achieving up to 100% sensitivity and 93% specificity for EOPE prediction [73].

Placental Protein 13 (PP-13), a placenta-specific dimeric protein [74], contributes to implantation, placental vascular development, and remodeling of maternal spiral arteries [5]. Usually, PP-13 levels rise across gestation, but reduced levels have been linked to subsequent PE [75]. Studies suggest that serum PP-13 combined with uterine artery pulsatility index can predict PE with growth restriction with reasonable accuracy [21,75]. Furthermore, first-trimester PP-13 alone [74], or combined with second-trimester uterine artery Doppler measurements [76], predicts EOPE more effectively than LOPE.

### 2.4. Monocytes and Macrophages in Preeclampsia

Immune system dysregulation is also a central contributor to the pathogenesis of PE. During a normal pregnancy, the maternal immune system undergoes significant adaptations that allow the fetus to survive immunologically throughout gestation [9]. These adaptations involve activation of endothelial monocytes and granulocytes as well as heightened systemic oxidative stress [8]. In PE, however, this response becomes exaggerated due to maladaptation in maternal–fetal immune interactions. Syncytiotrophoblast microparticles (STBMs), which are released into maternal circulation in greater amounts in PE than in normal pregnancies, are thought to play a key role in triggering systemic inflammation and endothelial damage. In fact, in EOPE, STBM levels were recorded to be markedly elevated compared to normal pregnancies. In LOPE, STBM levels did not differ significantly from those of controls [76]. This finding suggests that inflammatory activation and endothelial dysfunction may be more relevant to EOPE. Although many other immunological factors are modified in preeclamptic women, few have been systematically compared between EOPE and LOPE.

### 2.5. Cytokines and Inflammatory Imbalance

Proinflammatory cytokines are main contributors to functional and structural vascular changes, including oxidative injury and impaired vascular relaxation, thereby altering vascular integrity, tone, and coagulation [77]. Cytokine release is thought to be triggered when placental debris is phagocytosed by maternal monocytes and dendritic cells [78]. Tumor necrosis factor-α (TNF-α), one of the key proinflammatory cytokines, plays an important regulatory role by modulating the release of additional cytokines through complex feedback loops [79]. However, its role in PE remains controversial: some studies have reported elevated TNF-α levels [77,80], others reduced levels [79], and others found no difference [77,78,81]. Cackovic et al. observed significantly reduced TNF-α excretion in severe PE and suggested that earlier reports of higher levels might reflect methodological differences [79]. No studies have yet investigated whether TNF-α levels differ between EOPE and LOPE.

Interleukin-6 (IL-6), another proinflammatory cytokine secreted by activated leukocytes, influences vascular contractility and is linked to endothelial dysfunction and hypertension in PE. Elevated maternal IL-6 levels have been documented in several studies [82]. One study found that low IL-6 levels were associated with a reduced birth weight ratio, specifically in EOPE, whereas in LOPE IL-6 concentrations did not differ significantly from those of controls across birth weight categories [83].

Vascular cell adhesion molecule-1 (VCAM-1), an endothelial surface protein upregulated by inflammation [84], has also been studied in PE. Elevated VCAM-1 levels have been detected weeks before clinical onset [85] and are increased in both EOPE [86] and LOPE [16]. Raised VCAM-1 has also been linked to abnormal uterine artery Doppler findings. Interestingly, in LOPE with growth restriction, elevated sFlt1 and VCAM-1 levels have been observed, suggesting their joint contribution to intrauterine growth restriction. ICAM-1 levels are also higher in PE. Notably, in EOPE, the increases that have been recorded are greater than those in LOPE [87].

### 2.6. Oxidative Stress

Placental oxidative stress is considered an intermediate step in the development of PE. It is present when the amount of free radical products overpasses the body’s capacity for antioxidant defense [88]. Actually, the presence of oxidative stress in PE is supported by elevated nitrotyrosine levels, enhanced lipid peroxidation, trophoblast apoptosis, and decreased activity of critical antioxidant enzymes, as well as lower antioxidant levels [89]. Free radical activity within the placenta and maternal circulation is believed to trigger a cascade of mechanisms that weaken endothelial protection and promote the release of placental fragments [90,91]. Although its precise origin is not entirely clear, oxidative stress in PE is most often attributed to placental hypoxia [89].

The main production of free radicals in the preeclamptic placenta is performed in mitochondria. Another mechanism includes increased activity of xanthine oxidase and NAD(P)H oxidase. Reactive oxygen species generated by these pathways activate apoptotic mechanisms that can result in the release of syncytiotrophoblast fragments [88]. Oxidative stress is present in both entities, with varying degrees depending on the extent of trophoblast invasion impairment. Insufficient development of the cytotrophoblastic shell occurs early in pregnancy, leading to defective villous growth in EOPE, whereas oxidative stress tends to emerge later in pregnancy in LOPE.

NAD(P)H oxidase is a superoxide-generating enzyme that is expressed in placental trophoblasts, neutrophils, and endothelial cells. Its expression is upregulated in trophoblast and vascular smooth muscle cells from placentas of women with PE [91]. The resulting activation of NF-kB via NAD(P)H oxidase is thought to amplify the maternal inflammatory response by stimulating cytokine release and leukocyte activation. Women with EOPE show significantly higher total superoxide production than those with LOPE [91].

Lipid peroxidation, another hallmark of oxidative stress, produces lipid hydroperoxides as primary products [92]. If unchecked, lipid peroxidation disrupts cellular function and integrity [90,92]. In the platelets of women with PE, lipid peroxidation products have been found to be higher [90]. One study reported significantly higher levels of maternal erythrocyte malondialdehyde several weeks before the clinical manifestation of EOPE in the third trimester. However, no early increase in lipid peroxidation was observed in pregnancies complicated by LOPE [93]. It should be noted, however, that the sample size in this study was limited.

### 2.7. Innate Immune Pathways in Preeclampsia: NK Cells, Toll-like Receptors and Pentraxins

Uterine natural killer (uNK) cells represent a major immune population at the maternal–fetal interface in early pregnancy [70]. They play a crucial role in spiral artery remodeling and regulation of trophoblast invasion, thereby supporting placentation [94]. uNK cells also contribute to local cytokine production [95]. In preeclampsia (PE), these cells are more abundant in the placental bed [12]. Certain combinations of maternal killer immunoglobulin-like receptors (KIR) and paternal HLA-C haplotypes have been associated with increased susceptibility to PE [12,70]. Specifically, women lacking the protective KIR B haplotype appear at higher risk, particularly when carrying a fetus with a paternal HLA-C2 allele [94]. However, direct comparisons of uNK cell abundance or activity between early- and late-onset PE are still lacking.

Cytokine release by monocytes and trophoblasts can also be triggered by Toll-like receptors (TLRs), including TLR2 and TLR4, which sense endogenous danger signals generated by placental stress. The TLR2-mediated inflammatory pathway appears inhibited in PE [94,95,96], while studies on TLR4 gene polymorphisms have produced inconsistent findings: one found no association with PE or with EOPE versus LOPE [97], whereas another identified certain TLR4 allelic variants more frequently in women with a history of EOPE [98]. Activation of these innate receptors leads to downstream signaling through nuclear factor-κB (NF-κB), a central transcription factor regulating inflammatory and stress-response proteins [99]. NF-κB activation in preeclamptic placentas has been associated with neutrophil infiltration [100], leukocyte activation [80], and enhanced oxidative stress [101]. No direct comparative data exist on NF-κB expression between EOPE and LOPE.

Another family of innate immune mediators, the pentraxins, further illustrates the inflammatory–immune interplay in PE. Pentraxin-3 (PTX3), produced by vascular endothelial cells, monocytes, macrophages, and fibroblasts, modulates innate immunity by binding apoptotic antigens and preventing excessive immune activation [102]. Elevated maternal plasma PTX3 levels have consistently been associated with PE [101,102,103,104]. Importantly, PTX3 concentrations between 11 and 13 weeks are significantly higher in EOPE than in controls, whereas this difference is not observed in LOPE [103], suggesting a potential early biomarker distinguishing the two subtypes.

### 2.8. The Immune Maladaptation Hypothesis

While many studies support the immune maladaptation hypothesis, some epidemiological evidence has questioned its validity. Currently, maternal constitutional factors—particularly obesity—are thought to play a major role in the pathogenesis of LOPE, the most common phenotype of PE. Large Scandinavian and U.S. studies that argued against immune maladaptation primarily evaluated LOPE cases [105]. These studies showed that PE incidence in multiparous women increased with the number of years since their last delivery. After a 10-year interval, the risk was similar to that in primigravid women. The authors concluded that this rise was due to the prolonged birth interval rather than the introduction of a new partner (the primipaternity effect). However, more recent findings suggest that extended intervals may also involve immune maladaptation.

Paternal antigen-specific regulatory T (PAS-Treg) cells persist after pregnancy but have a limited lifespan. Their numbers decrease after about 10 years, making multiparous women with long interpregnancy intervals as susceptible to PE as primigravidas [106]. Immune maladaptation is thought to contribute to shallow trophoblast invasion of spiral arteries, resulting in placental dysfunction and fetal growth restriction (FGR), which is more typical of EOPE.

From a clinical perspective, recognition of immune maladaptation has several clinical implications. First, longer exposure to paternal sperm before conception appears partially protective against EOPE. Thus, although condom use is still advised to prevent sexually transmitted infections, sustained sperm exposure within a stable relationship may reduce PE risk [80,81]. Moreover, multiparous women with a new partner should be managed similarly to primigravidas according to the primipaternity concept [107,108]. For this reason, both primigravidas and multiparas with a new partner should be asked about the duration of sexual cohabitation with the current father [109,110]. Finally, pregnancies conceived through donor insemination, oocyte donation, or embryo donation carry an increased risk of hypertensive disorders [109,110].

Notably, the “new father” effect (primipaternity) is associated not only with a higher risk of PE but also with lower infant birthweight. Multiparous women with a new partner often display similar birthweight outcomes to primigravid women [111]. Historically, primiparous women have been observed to deliver slightly lighter babies than multiparas [111]. Recent analyses show that even after adjusting for PE, smoking, alcohol use, and maternal BMI, primigravidas and multiparas with a new partner deliver infants 100–150 g lighter on average [111,112]. This finding supports the theory of the “maternal–fetal graft paradox.” This means that the first pregnancy constitutes a less efficient immunological adaptation. In subsequent pregnancies with the same partner, maternal tissues are already primed, facilitating better outcomes [111,112].

In the 2018 workshop on PE immunology, experts emphasized that with a hemochorial placenta, women face a potentially intense immunologic “attack” at the feto-maternal interface. Inadequate immune tolerance may result in PE and IUGR [113]. In this context, epidemiologic data recorded an inverse relation between the duration of sexual cohabitation and the risk for PE. This finding further supports the role of prolonged sperm exposure in successful implantation [112,113]. This aligns with the fact that human females are exposed to semen repeatedly before conception, unlike most mammals. From an evolutionary perspective, inducing PAS tolerance through sustained exposure may improve embryo implantation and survival in long-term relationships.

The relatively high prevalence of PE in humans may represent an evolutionary disadvantage compared with other mammals. Historically, eclampsia accounted for at least 1% of all births, even in developed countries up until the mid-20th century [113]. Robillard et al. hypothesized that the existence of PE as a clinical syndrome reflects a major reproductive burden requiring human adaptation [113,114]. Human pregnancies involve unique features: (1) PE and eclampsia occur only in humans, and (2) trophoblast invasion in humans extends deeply into the myometrium. This invasion allows semi-allogeneic trophoblast cells to replace maternal endothelial cells in spiral arteries, disrupting the vascular smooth muscle layer. Such intense maternal–fetal cellular interaction requires a higher degree of immune tolerance than in other species.

Robillard et al. [17,113,114] further suggested that a major difference between humans and other mammals is the large fetal brain, which demands about 60% of total fetal nutrition during the rapid development of the second and third trimesters. This high demand necessitates deep trophoblast invasion and high maternal blood flow to the intervillous space. Such invasion, however, could only evolve through significant maternal–paternal immunogenetic compromises.

The idea that PE has an immunological origin dates back to the early 20th century [115,116]. In the 1950s, Medawar introduced the concept of the fetus as a “semi-allograft” [117]. Since then, fetal implantation has been viewed as a maternal immune process mediated by T cells recognizing paternal alloantigens. Initially, the importance of innate immunity was overlooked, but in the late 20th century, it became clear that innate immune mechanisms are fundamental in reproductive immunology.

### 2.9. Interaction Between Immunologic Alterations and the Placental Metabolic Syndrome in PE

In recent years, the association between PE and metabolic syndrome has become increasingly evident [118,119]. One proposed mechanism involves the role of inositol phosphoglycans (IPGs) in PE pathogenesis. An imbalance between circulating angiogenic factors and insulin second messengers, such as IPGs, may contribute to the “placental” insulin resistance observed in PE [120,121]. IPGs are mediators of insulin’s trophic effects, enhancing protein synthesis, cell growth, differentiation, and survival, and are derived from the fetal or placental unit [122].

A lipidic form of IPGs, which exhibits proinflammatory and endotoxin-like properties, can cross into maternal circulation due to increased placental membrane permeability. This occurs because of immunological changes leading to a thinner glycocalyx and reduced tight junctions. Such lipidic IPGs, including the P-type, may contribute to endothelial injury and the development of atherosclerosis [121]. Placental ischemia, inflammation, and reperfusion damage also promote glycocalyx shedding and loss of tight junction integrity, further facilitating leakage of IPGs into maternal blood [121,123].

Importantly, IPGs have been detected in maternal urine several weeks before the clinical onset of PE in both EOPE and LOPE [124,125]. Along with other urinary biomarkers, IPGs could therefore serve as inexpensive and practical tools for early diagnosis, particularly in low-resource settings [99]. While the sFlt-1/PlGF ratio is widely used for EOPE prediction [126,127], urinary IPGs may be more useful for identifying LOPE.

### 2.10. Molecular Insights into Early- and Late-Onset Preeclampsia

Admati et al. aimed to investigate molecular differences between the various presentations of PE [93]. We conducted an unbiased single-cell transcriptomic survey comparing all placental cell types from cases with early- and LOPE pregnancies with those from matched controls at the same gestational age. This single-cell approach enabled the analysis of gene expression patterns across different cell classes and the identification of any dysregulation associated with the disease. In EOPE, our findings showed a widespread dysregulation of gene expression across all cell types. Notably, a significant imbalance in FLT1 and PlGF has been observed. This imbalance was observed in the syncytium of early PE subjects, indicating a cell-autonomous dysregulation of FLT1 and PlGF transcription. The stromal cells and vasculature were characterized by an inflammatory, stress, and antiangiogenic environment. Furthermore, the placental immune niche played a crucial role in driving inflammation in EOPE. We showed that both fetal-origin Hofbauer cells and maternal-origin triggering receptor expressed on myeloid cells 2 (TREM2) played significant roles in this process. In contrast, local cells of the adaptive immune system were largely unaffected. In comparison, LOPE had minimal impact on placental cellular function, with preserved angiogenic balance. Taken together, this provides molecular support for the existence of two distinct types of PE. One involves pathologic angiogenic−antiangiogenic balance, whereas the second is not placental but rather a maternal cardiovascular-based maladaptation to pregnancy. As such, the two phenotypes require different preventative and interventional management approaches [128].

### 2.11. Risk Factors

Risk factors of EOPE include preexisting hypertension, autoimmune diseases (e.g., lupus, antiphospholipid syndrome), thrombophilias, primi paternity, prolonged interbirth intervals, and previous history of PE. There is, however, some controversy regarding these observations, given that some studies used mainly the term PE (Type II), where immune maladaptation is unlikely to play a significant role [129]. These findings contribute to our understanding of nulliparity as a substantial risk factor for pre-eclampsia and highlight the greater robustness of placentation [113,114] and other aspects of pregnancy and lactation in women who have given birth compared with those who have not [130,131]. LOPE risk factors are associated with obesity, diabetes mellitus, advanced maternal age, and multiparity.

### 2.12. Ultrasonographic Markers and Diagnostic Role of Imaging in Preeclampsia

Ultrasonography is central to the evaluation of uteroplacental and fetal circulations in PE. Uterine artery Doppler often shows elevated pulsatility index (PI > 95th centile) and bilateral notching in EOPE, reflecting impaired spiral-artery remodeling; these findings are less typical in LOPE [7,18]. Umbilical artery Doppler abnormalities (elevated PI, absent/reversed end-diastolic flow in severe cases) indicate placental vascular resistance and correlate with fetal growth restriction, a hallmark of EOPE, whereas LOPE typically shows normal flow dynamics with a predominant maternal cardiovascular contribution [7]. Middle cerebral artery (MCA) PI and cerebroplacental ratio (CPR) (MCA-PI/UA-PI) aid hypoxia risk assessment—CPR < 5th centile signals brain-sparing and adverse perinatal risk. Integrating Doppler indices with angiogenic biomarkers (e.g., sFlt-1/PlGF) enhances prediction and short-term risk stratification, particularly near term [100]. Exploratory markers (e.g., ophthalmic artery Doppler) have been investigated as adjuncts but are not yet standard of care [9,37,38,132]. A summary of the main ultrasonographic and Doppler markers used to assess preeclampsia, along with their distinguishing patterns in EOPE and LOPE, is presented in Table 2.

### 2.13. Prevention of Preeclampsia

To date, we have strong evidence that EOPE, the dangerous but relatively rare form of PE in terms of maternal and fetal morbidities, can be prevented in up to 62% of cases by aspirin 150 mg/d when started before 16 weeks’ gestation [131]. Taking aspirin from the early pregnancy period may reduce the degree of shallow placentation (i.e., a sign of PE) and prevent the development of the disease. However, what about the far more common problem of LOPE? Although typically associated with a benign perinatal outcome, this entity may be a more important cause of maternal morbidity and mortality than EOPE. Recently, two different teams from different parts of the world have reported that late-onset PE (and much less EOPE) is primarily and specifically associated with a linear increase in maternal BMI [133,134]. Further research is urgently required to properly understand the main drivers and pathways of how cardiometabolic syndrome leads to LOPE [121]. Optimizing pre-pregnancy weight represents a crucial primary preventive strategy. Recently, we demonstrated that having a high BMI does not automatically translate into a high risk of term PE.

In a large population cohort study, we showed that patients with obesity could decrease their risks by optimizing their GWG [135,136]. Extensive randomized controlled studies are required to evaluate whether these observational data can be replicated in a prospective trial. In summary, our understanding of the pathophysiology of PE has undergone significant improvement. Immune maladaptation is involved in the development of EOPE. Low-dose aspirin reduces the risk of early-onset PE, but not the risk of LOPE. In contrast, obesity and metabolic syndrome are associated with LOPE, but this is not an established method for their prevention. Broad and sustained public education toward healthy pre-pregnancy weight and possibly subsequently optimizing GWG may have the potential to reduce LOPE significantly.

An overview of the differences between early- and late-onset preeclampsia concerning the key clinical and laboratory characteristics, the screening process, the risk factors, and pregnancy surveillance is provided in Table 3.

### 2.14. Insights Gained from First Pregnancies

At the maternal–fetal interface, dNK cells appear to keep a “memory” of prior pregnancies [112,130,137,138]. These pregnancy-trained dNK cells display a higher capacity for growth factor release, immune regulation, and production of chemokine ligands and receptors, supporting effective placentation [130]. The maternal heart also has a “memory” and appears to “remember” pregnancy, resulting in improved cardiovascular adaptation in subsequent pregnancies [139,140]. There is growing evidence of immune system involvement in the development of PE, particularly in women with preexisting autoimmune disorders [141]. Additionally, compatibility between maternal immune receptors expressed on natural killer cells and paternal antigens expressed on trophoblasts appears to play a significant role [142].

Furthermore, untried maternal immunologic tolerance, as observed in pregnancies with a new father or with donor semen in insemination, increases the risk of pre-eclampsia. A large prospective study demonstrated that a short duration of sexual relationship was more common among women with features of placental insufficiency [143]. Conversely, a longer duration of cohabitation before conception seems to have a protective effect against PE.

To summarize, PE can arise through 2 pathways. Numerous studies have demonstrated that gestational cardiac adaptations, both in normal and preeclamptic women, begin early in pregnancy, persist after delivery, and remain detectable in subsequent pregnancies. Research into PE has consistently shown that the placenta is deeply involved in both the formation of the maternal–fetal interface and the maternal endothelial and cardiac adjustments that support pregnancy [5]. Immune activation in pregnancy creates memories of pregnancy in the uterine and cardiovascular systems, which contribute to the greater robustness of subsequent pregnancies, including reduced risk of PE of both types [112,130,137].

### 2.15. The Impact of the Fetus on Maternal Cardiac Remodeling

Maternal cardiac function influences fetal development through cardiac remodeling, reflected in changes such as increased aortic root diameter, larger left atrial diameter, greater left ventricular mass, and elevated blood pressure—even in women with otherwise uncomplicated pregnancies [144]. More recently, Giorgione et al. demonstrated a correlation between sFlt-1, PlGF, and their ratio and maternal echocardiographic indices in patients with hypertensive disorders of pregnancy. Their results lend support to the notion that angiogenic imbalance during pregnancy is associated with adverse maternal cardiovascular outcomes in pregnancy and beyond [145]. Pregnancy without placental PlGF-modulated remodeling produces measurable long-term injury to the maternal cardiovascular system. These studies in humans and murine models highlight that low PlGF, even in the absence of clinical PE, has long-term deleterious effects, serving as further proof of the imperative role of the placental PlGF in gravid remodeling of the cardiovascular system [144,146,147]. Indeed, maternal cardiac maladaptation has been observed across various parameters. In a study comparing uterine artery Doppler and echocardiographic measures among preeclamptic women and normotensive controls, the EO preeclamptic group showed reduced ventricular wall thickness and diameters, lower CO and apparent underfilling, and signs of pressure overload and concentric hypertrophy. Those with LO disease exhibited enlarged ventricles, high CO, ventricular hypertrophy, and signs of overfilling without pressure overload. EO- and LO-PE also differed in terms of PVR, with high PVR in EO disease and low PVR in LO disease [148]. It has been shown that increased Doppler indices of the ophthalmic arteries [109,112] in the late first trimester are predictive of the development of PE [24,26,27,34] and, measured in the third trimester [24], are also predictive of the development of PE in the next 3 weeks [27]. Considering the pervasive maternal cardiovascular adaptational changes that occur during pregnancy and the disorders in these adaptations observed in PE, it is unsurprising that these extend to divergent adaptations in the peripheral vasculature, such as the ophthalmic arteries.

## 3. Emerging Mechanisms and Gene Therapy Perspectives

Emerging evidence suggests that gene therapy may offer a new approach to addressing placental dysfunction in PE. Recent developments include the FDA Fast Track designation of CBP-4888, an siRNA therapeutic that downregulates sFLT-1 expression in the placenta. Strategies under investigation range from gene suppression (using siRNA and miRNA) and gene editing to gene overexpression. Despite the high interest that viral and non-viral vectors are drawing for use, non-viral systems are preferred because of safety considerations. Research is moving toward placenta-specific delivery, with nanoparticles emerging as valuable tools. Key therapeutic targets include sFLT-1, VEGF, IGF, microRNAs, and RAAS. Challenges such as model variability, safety, ethical considerations, and the complex pathology of PE remain, but CBP-4888 marks an important milestone in the field [149].

## 4. Conclusions

EOPE and LOPE represent two distinct forms of the disease with different pathophysiological origins, risk factors, clinical manifestations, and outcomes. EOPE develops before 34 weeks of gestation and is primarily driven by abnormal placentation [7,10,11,12,13,14], and this subtype is associated with a higher risk of severe maternal and fetal complications [123,126]. In contrast, LOPE, presenting at or after 34 weeks, is more often linked to maternal cardiovascular and metabolic predisposition, following a milder course with better perinatal outcomes [112,131]. EOPE typically manifests with a sudden onset and rapid progression, whereas LOPE tends to develop more gradually. In terms of incidence, LOPE is more common than EOPE, accounting for the majority of preeclampsia cases [123]. Both forms require careful monitoring and timely management to minimize complications. Current treatment focuses on blood pressure control, seizure prophylaxis, and delivery of the fetus once maternal or fetal safety can no longer be ensured [7,14]. Recognizing and understanding these mechanistic and clinical distinctions is crucial for accurate diagnosis, effective management, and prevention of both acute and long-term complications associated with preeclampsia. Future studies, molecular-oriented for therapeutic targets and preventive strategies, will help optimize management.

## Figures and Tables

**Table 1:** Definitions of preeclampsia.

Guideline/Source	Definition of Preeclampsia	Proteinuria/Other Criteria	Additional Features/End-Organ Dysfunction
Traditional (WHO/Classic)	New-onset hypertension after 20 weeks (≥140/90 mm Hg)	Proteinuria ≥ 300 mg/24 h or ≥2 + dipstick	–
ACOG (2019)	New-onset hypertension after 20 weeks (≥140/90 mm Hg on 2 occasions, ≥4 h apart)	Proteinuria ≥ 300 mg/24 h or PCR ≥ 30 mg/mmol	In absence of proteinuria: renal insufficiency (SCr > 97 µmol/L), hepatic involvement (AST/ALT > 2 × ULN), thrombocytopenia (<100,000/µL), pulmonary edema, neurological symptoms
ISSHP-M (2021)	New-onset hypertension (≥140/90 mm Hg on ≥2 occasions)	Proteinuria ≥ 300 mg/24 h or PCR ≥ 30 mg/mmol	At least 1: renal insufficiency (SCr ≥ 90 µmol/L), hepatic involvement (AST/ALT > 40 IU/L), thrombocytopenia (<150,000/µL), neurological symptoms (visual disturbance, clonus, etc.)
ISSHP-MF (2021)	As above + evidence of uteroplacental dysfunction	–	Fetal growth restriction (EFW < 10th centile with abnormal Dopplers) or fetal death
ISSHP-MF-AI (2021)	As above + biochemical evidence of angiogenic imbalance	–	sFlt-1/PlGF ratio > 95th percentile or PlGF < 5th percentile

Abbreviations: PCR = protein-to-creatinine ratio; SCr = serum creatinine; ULN = upper limit of normal; EFW = estimated. fetal weight.

**Table 2:** Key Ultrasonographic and Doppler Markers in Preeclampsia.

Parameter/Marker ^Ref^	Typical Findings in EOPE	Typical Findings in LOPE	Clinical Interpretation
**Uterine artery Doppler (PI, notching)** ^[15,16,25]^	PI > 95th percentile; bilateral early diastolic notches frequent	Usually normal or mildly elevated PI; notching uncommon	Reflects impaired spiral artery remodeling and uteroplacental hypoperfusion; predictive of EOPE
**Umbilical artery Doppler (PI, EDF pattern)** ^[15,18,25]^	Elevated PI; possible absent/reversed end-diastolic flow (AEDF/REDF) in severe cases	Normal or mildly elevated PI; normal EDF	Indicates increased placental vascular resistance and fetal growth restriction
**Middle cerebral artery (MCA) PI** ^[15,25,39]^	Decreased (<5th percentile) due to brain-sparing response	Often within normal range	Reflects fetal adaptation to hypoxia; part of cerebroplacental ratio (CPR) assessment
**Cerebroplacental ratio (CPR = MCA-PI/UA-PI)** ^[25,39,126]^	<5th percentile (abnormal)	Typically normal	Sensitive marker of fetal compromise and adverse perinatal outcome
**Placental morphology (B-mode)** ^[13,14,15]^	May show thickened or lobulated placenta, but nonspecific	Usually normal appearance	Morphologic changes alone are not diagnostic; Doppler assessment is essential
**Ophthalmic artery Doppler (maternal)** ^[38,39,40,132]^	Possible increased resistance index, indicating impaired maternal vascular adaptation	Near-normal hemodynamic profile	Experimental marker for maternal endothelial function; not yet in clinical use
**Integration with angiogenic biomarkers (sFlt-1/PlGF)** ^[39,126]^	Markedly increased ratio; abnormal values correlate with EOPE and adverse outcomes	Mild or moderate increase	Enhances short-term prediction and risk stratification, especially near term

PI, Pulsatility Index; EDF, End-Diastolic Flow; AEDF, Absent End-Diastolic Flow; REDF, Reversed End-Diastolic Flow; CPR, Cerebroplacental Ratio.

**Table 3:** Preeclampsia classification based on pathophysiology, clinical/laboratory characteristics and treatment strategies.

	Early Onset Preeclampsia (<34 Week)	Late Onset Preeclampsia (≥34 Week)
Screening	Maternal factors, mean arterial pressure, uterine artery Doppler, and PlGF	--------
Risk factors	Nulliparity Previous preeclampsia Diabetes IVF without corpus luteum IVF with donor eggs Antiphospholipid syndrome Molar pregnancy Fetal conditions	Nulliparity Previous preeclampsia Diabetes IVF without corpus luteum IVF with donor eggs Obesity Chronic hypertension Chronic kidney disease
Common clinical and laboratory characteristics	Fetal growth restriction sFlt-1/PlGF ↑↑↑ Cardiac output ↓ Peripheral vascular resistance ↑	Macrosomia/twins and multiples sFlt-1/PlGF ↑ Cardiac output ↑ Peripheral vascular resistance ↓
Pregnancy surveillance	Clinical parameters Laboratory studies (includingsFlt-1/PlGF) Doppler studies Estimated fetal weight Maternal cardiac studies	Clinical parameters Laboratory studies (includingsFlt-1/PlGF) Estimated fetal weight
Preventative strategies	Exercise duration » 140 min/week Aspirin Aspirin + LMWH (in presence of antiphospholipid antibody) Calcium administration Progesterone support in IVF pregnancy?	Exercise duration ≥ 140 min/week Glycemic control Weight control and reduction Prevention of multiple pregnancy
Defined strategies	Exercise NO donors, calcium channel blockers, fluid support aimed at vasodilation Timed delivery	Exercise Alpha/beta blockers Timed delivery
Future strategies	sFlt-1ligands siRNA-based therapy Plasmapheresis Antioxidants	------------

IVF: in vitro fertilization; LMWH: low molecular weight heparin; NO: nitric oxide; PlGF: placental growth factor; sFlt-1: soluble Fms-like tyrosine kinase-1; siRNA: small interfering RNA.

## Data Availability

No new data were created or analyzed in this study. Data sharing is not applicable to this article.

## Abbreviations

The following abbreviations are used in this manuscript:

EGF	Multidisciplinary Digital Publishing Institute
EOPE	Early onset preeclampsia
HELLP	Hemolysis, Elevated Liver enzymes and Low Platelets
HLA	human leukocyte antigen
FGR	fetal growth restriction
IL-	interleukin-
IPGs	inositol phosphoglycans
KIR	killer immunoglobulin-like receptors cells
NF-kB	Nuclear factor-kB
LOPE	Late onset preeclampsia
PE	Preeclampsia
PlGF	Placental Growth Factor
PTX3	Plasma pentraxin 3
RAAS	renin–angiotensin–aldosterone system
sEng	soluble endoglin
sFlt1	soluble Fms-like tyrosine kinase
STBMs	Syncytiotrophoblast microparticles
sVEGFR-1	soluble Vascular endothelial growth factor receptor-1
TGF-β	transforming growth factor-β
TNF-α	tumor necrosis factor-α
TLRs	toll-like receptors
TREM2	triggering receptor expressed on myeloid cells 2
VCAM	Vascular cell adhesion molecule
VEGF	Vascular Endothelial Growth Factor
VEGFR-1	Vascular endothelial growth factor receptor-1
